# Advanced cervical stump cancer after laparoscopic subtotal hysterectomy: a case report of imaging, laparoscopic staging and treatment approach

**DOI:** 10.1186/s12905-023-02428-7

**Published:** 2023-05-23

**Authors:** Dimitrios Andrikos, Argyrios Andrikos, Antoine Naem, Olga Ebertz, Rajesh Devassy, Rudy Leon De Wilde, Michael Khamou, Harald Krentel

**Affiliations:** 1 Department of Gynecology, Obstetrics and Gynecologic Oncology, Academic Teaching Hospital Bethesda, Duisburg, Germany; 2 Faculty of Mathematics and Computer Science, University of Bremen, Bremen, Germany; 3 Centre of Excellence in Gynecological Minimal Access Surgery and Oncology, Dubai London Clinic & Specialty Hospital, Dubai, 3371500 United Arab Emirates; 4 University Hospital for Gynecology, Pius-Hospital Oldenburg University Medicine, Oldenburg, Germany; 5 Department of Radiology, Academic Teaching Hospital Bethesda, Duisburg, Germany

**Keywords:** Cervical Cancer, Supracervical hysterectomy, Cervical dysplasia, Laparoscopic lymphadenectomy, Human papilloma virus

## Abstract

**Background:**

Advanced cancer of the cervical stump, occurring years after a laparoscopic supracervical hysterectomy (LASH), is a rare but serious clinical condition. Many patients who undergo a LASH are unaware of this possible complication. Upon diagnosis of advanced cervical stump cancer, a holistic approach including imaging, laparoscopic surgery and multimodal oncological therapy is required.

**Case presentation:**

A 58-year-old patient presented to our department with the suspicion of advanced cervical stump cancer eight years after LASH. She reported pelvic pain, irregular vaginal bleedings and irregular discharge. Gynaecological examination revealed a locally advanced tumor of the uterine cervix with suspicion of infiltration of the left parametria and bladder. After thorough diagnostic imaging and laparoscopic staging, the tumor stage was determined as FIGO IIIB and the patient was treated with combined radiochemotherapy. The patient presented with tumor recurrence 5 months after the completion of therapy and she is currently being treated with multichemotherapy and immunotherapy regimens as palliative treatment.

**Conclusion:**

Patients should be made aware about the risk of cervical stump carcinoma after LASH and the necessity for regular screening. Cervical cancer after LASH is often diagnosed at advanced stages and the treatment requires an interdisciplinary approach.

## Background

Laparoscopic supracervical hysterectomy (LASH) is a safe and feasible minimally invasive approach with good outcomes when performed to patients with benign gynaecological diseases like uterine fibroids and adenomyosis [1, 2]. LASH is performed only under certain condititions such as a normal Papanicolaou Smear (PAP I), no clinical evidence of uterine or cervical dysplasia, no evidence of retrocervical endometriosis, patient’s desire to preserve the cervix and willingness to undergo future cervical cancer screening [3]. It is also performed as an emergency peripartum hysterectomy in case of obstetric complications such as severe uterine atony [4]. Perioperative outcomes favor subtotal hysterectomy over total hysterectomy. However patients who undergo LASH present occasionally with cyclical vaginal bleeding in about 6% of cases and could develop cervical stump cancer. The last accounts for 1.6–4.4% of the overall cervical cancer cases [5].

Cervical cancer screening is very heterogeneous internationally. Germany’s actual guidelines recommend an annual PAP smear for women aged 20–34 years old. In women older than 35 years, a combined HPV and PAP Tests are recommended every 3 years. For women who have had a supracervical hysterectomy, the exact same screening protocol should be followed.

After the development of cervical cancer, therapy is adapted to the stage of the disease according to Tumor, Nodes, Metastases (TNM) staging and International Federation of Gynaecology and Obstetrics (FIGO) classification. In stages from FIGO IA to FIGO IB2 and occasionally in FIGO IIA, surgery is recommended. On the other hand, primary combined radiochemotherapy is administered in FIGO stages IIB, III and IV. Additionally, FIGO IB3 tumors or smaller tumors with risk factors such as lymphovascular invasion, deep stroma infiltration or lymph nodes metastases are treated with combined therapy as well [6]. Neoadjuvant chemotherapy followed by radical surgery could be an alternative to combined radiochemotherapy in some cases, but some factors such as parametrial infiltration should be considered [7]. Upon diagnosis of distant metastases, palliative chemotherapy combinated with angiogenesis inhibitors was until recently the standard of care. According to some promising analyses, immunotherapy is now being administered additionally to the aforementioned therapies to patients with a combined positive score (CPS > 1) after immunhistochemistry testing for Programmed Death Ligand-1 (PDL-1) expression [8, 9]. The management of cervical stump carcinoma usually follows the same aforementioned principles.

Patients who undergo LASH should be well-informed about the possible complications and the need for a future cervical cancer screening before performing a subtotal hysterectomy. In this paper, we report the imaging and treatment approaches of a case of advanced uterine cervical stump carcinoma after laparoscopic subtotal hysterectomy.

## Case presentation

### Diagnostic challenges in advanced cancer of the uterine cervix after LASH

**Fig. 1:**
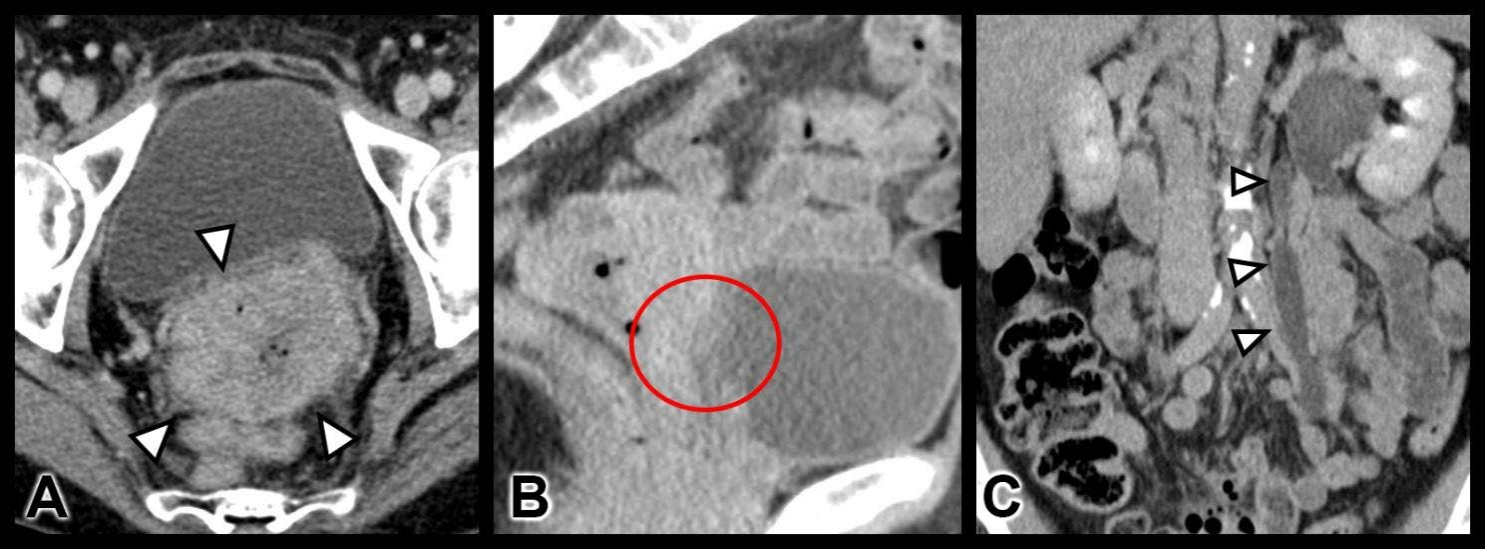
** A)** Axial oblique contrast-enhanced CT scan shows a large heterogeneous cervical mass abutting the urinary bladder without definite invasion. **B)** Sagittal contrast-enhanced CT scan shows the large exophytic cervical mass extending anteriorly into the vaginal vault. **C)** Coronal oblique contrast-enhanced CT scan shows left-sided stage I hydronephrosis with dilated ureter due to tumoral invasion

A 58-year-old patient presented to our certified tertiary department of gynaecological oncology with the suspicion of an advanced cancer of the cervical stump 8 years after LASH. She reported pelvic pain, irregular vaginal bleedings and irregular vaginal discharge. In 2013, the patient underwent a LASH for symptomatic uterine fibroids in another hospital. No cervical cancer screening was performed after the operation. Gynaecological examination revealed a locally advanced tumor of the uterine cervix with suspicion of infiltration of the left parametria and bladder. Abdominal ultrasound showed a stage I hydronephrosis of the left kidney with the beginning of hydronephrosis in the right kidney. Biopsy revealed a poorly differentiated squamous cell carcinoma (Grade 3) of the uterine cervix. Computed tomography (CT) scan demonstrated a cervical tumor with a maximum diameter of 6.4 cm (Fig. 1A and B). It was also remarkable for pelvic lymphadenopathy, infiltration of the tumor into the posterior vaginal vault, and compression of the left ureter with subsequent hydronephrosis. Infiltration of the posterior bladder wall was also suspected (Fig. 1C). No free fluid in the pouch of Douglas or distal metastasis were observed.

**Fig. 2:**
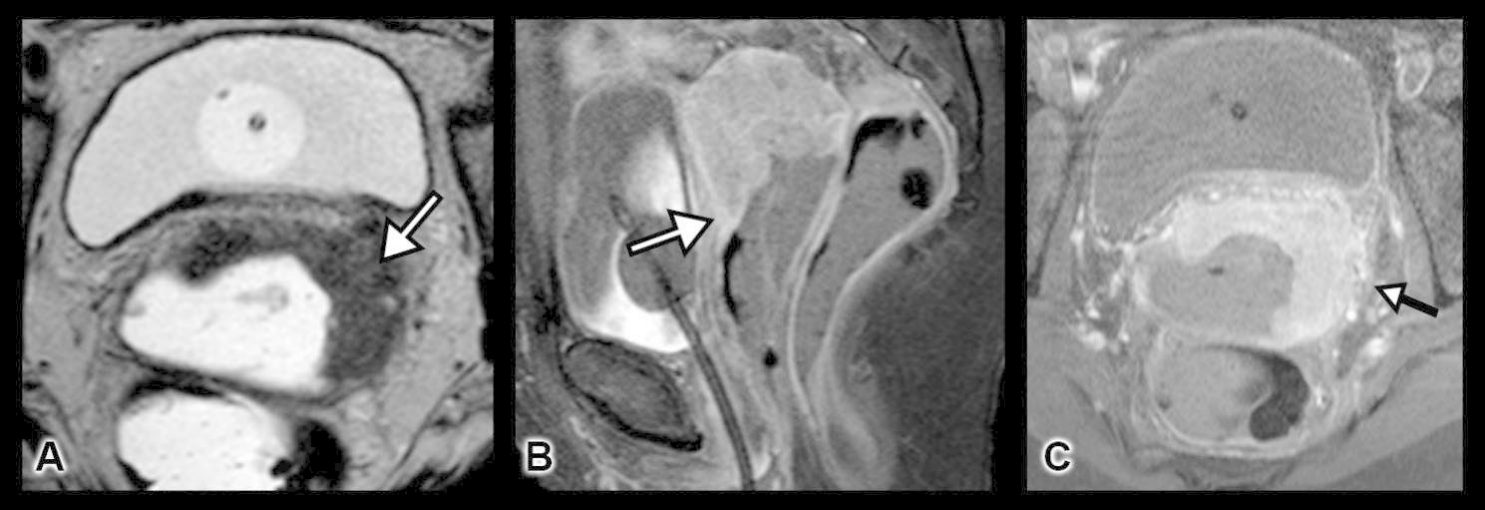
** A)** Axial T2-weighted MRI shows a large cervical mass with enrcirclement of the left ureter and subtle mucosal edema of the urinary bladder. Vaginal and rectal gel instilled prior to examination. Foley catheter in situ. **B)** Sagittal contrast-enhanced T1-weighted MRI with fat saturation shows a homogenously enhanced cervical mass expanding into the vaginal vault. **C)** Axial oblique contrast-enhanced T1-weighted MRI with fat saturation shows the circular tumor growth of the cervical mass. In this patient a bilateral parametrial invasion is present (Right side not shown)

A preoperative Magnetic Resonance Imaging (MRI) was performed to assess the degree of bladder and surrounding organs involvement. It revealed a tumor of 58 × 46 × 37 mm infiltrating the vaginal fornix, mucosal edema of the bladder, and encirclement of the left ureter. The pelvic lymph nodes were suspicious. No peritoneal infiltration was indicated (Fig. 2).

After completion of diagnostic imaging, we defined the tumor preoperatively as a cT4a cervical cancer with possible infiltration of the bladder muscularis. Mucosal infiltration of the bladder was later excluded by a diagnostic cystoscopy during which a ureteral stent on the left side was implanted.

### Therapeutic challenges in advanced cancer of the uterine cervix after LASH

**Fig. 3:**
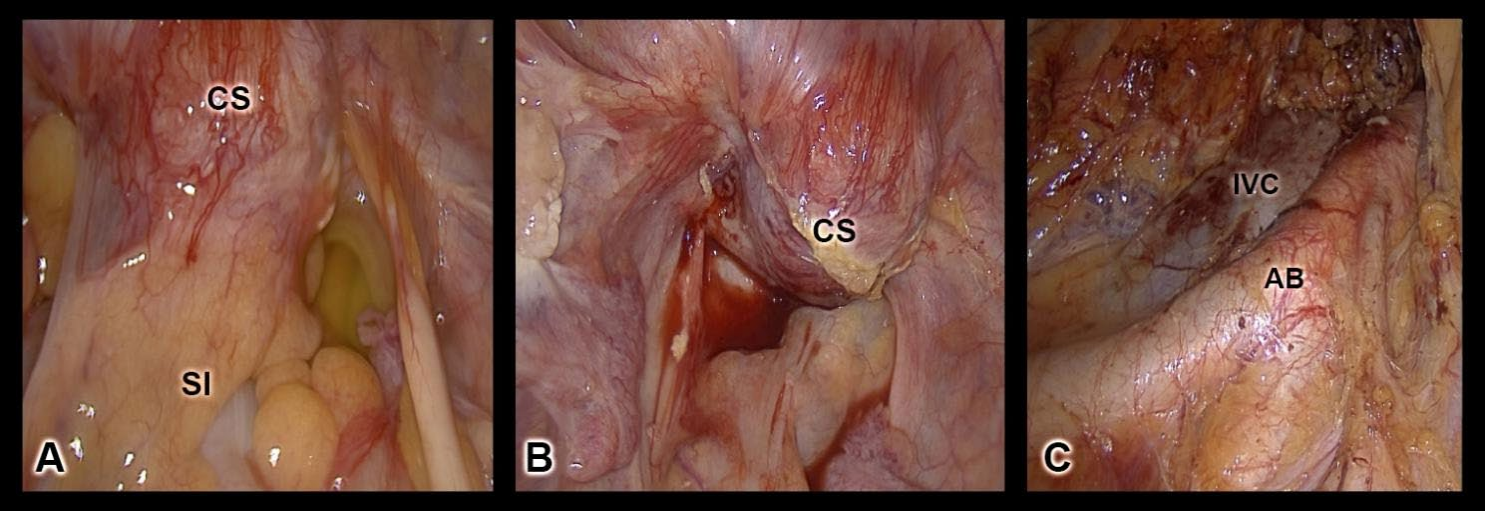
** A)** Laparoscopic initial view shows bowel adhesions to the uterine cervix after LASH. No peritoneal infiltration. **B)** Laparoscopic view of uterine cervix after adhesiolysis, hyperemia of the tumor region. **C)** Laparoscopic view of the aorta and the two main iliac arteries after lymphadenectomy. CS: Cervical Stump, SI: Small Intestines, IVC: Inferior Vena Cava, AB: Aortic Bifurcation

After complete presurgical assessment and discussion of the appropriate therapeutical approach in our interdisciplinary oncological board meeting, we performed a laparoscopy with peritoneal lavage, complete adhesiolysis, peritoneal biopsies, appendectomy, bilateral salpingo-oophorectomy, pelvic and paraaortic staging lymphadenectomy. Intraoperative findings included intraperitoneal adhesions involving the intestines and a cervical stump carcinoma infiltrating the left pelvic sidewall, both parametria, the anterior and posterior vaginal fornices. The infiltration of the peritoneum of the pouch of Douglas was also suspected. However, intraoperative biopsies and frozen sections did not demonstrate malignant invasion. More extensive invasion of the left parametrium was noted. However, the bladder and rectum were not invaded by the tumor. As the clinical stage was defined as FIGO IIIB, a staging paraortic lymphadenectomy was performed to define the postoperative radiation field. A sampling pelvic lymphadenectomy was performed as well. Seven lymph nodes of the left pelvic wall, four of the right pelvic side wall and 13 paraortic lymph nodes were removed without any histological tumor infiltration (Fig. 3).

Postoperative recovery of the patient was uneventful. After incorporating the histological report, the tumor was classified as FIGO IIIB cervical cancer.

**Fig. 4:**
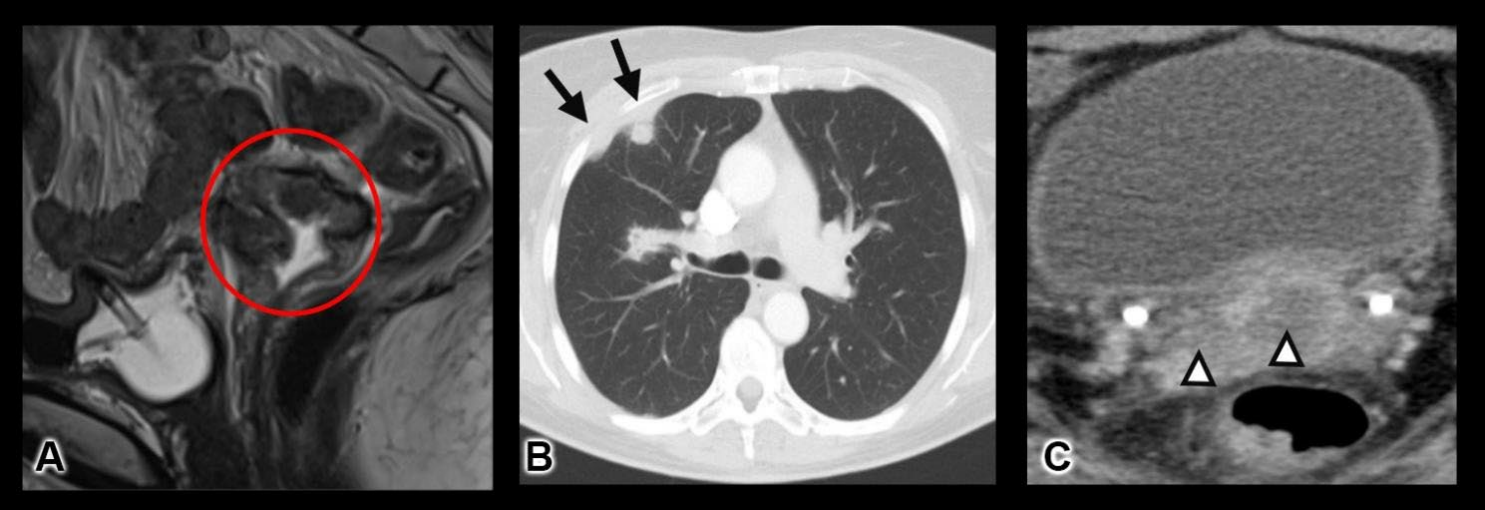
** A)** Sagittal T2-weighted MRI after radiochemotherapy shows satisfactory decrease in tumor size. **B)** Axial contrast-enhanced CT scan shows a solid nodule with spiculation in the posterior segment of the right upper lobe. Moderate pleural thickening with a pleural-based nodule in the anterior segment of the same lobe. **C)** Axial contrast-enhanced CT scan shows the cervical tumor with areas of encapsulated necrosis

A multi-disciplinary treatment approach was suggested with direct involvement of our in-house radiotherapy and oncology departments after discussing the case in the interdisciplinary oncologic meeting. We suggested a combined radiochemotherapy consisting of percutaneous radiation as well as vaginal after-loading with 6 Cycles of Cisplatin 40 mg/m^2^ weekly as a radiosensitizer. After implantation of a venous port system the patient started with treatment on January 2022 and finished on March 2022. Most of the radiochemotherapy required the hospitalization of the patient due to inappetence, fatigue, nausea and derailed diabetes mellitus type 1. Post-therapeutic MRI revealed tumor regression with adequate tumor response (Fig. 4A).

### Tumor recurrence

Five months after completing the treatment with combined radiochemotherapy, the patient suffered a tumor recurrence as depicted in the CT scan. It showed local tumor progression with associated necrosis as well as distant pleural and pulmonary metastases (FIGO IVb) (Fig. 4B C). Immunhistochemistry testing for Programmed Death Ligand-1 (PDL-1) expression revealed a combined positive score of 35. We suggested a combined multichemotherapy with immunotherapy consisting of intravenous Carboplatin/ Paclitaxel/ Bevacizumab/ Pembrolizumab every 3 weeks according to local and international guidelines for systemic treatment of first-line metastatic cervical cancer.

## Discussion and conclusions

Supracervical hysterectomy is a routine gynaecological surgery with a low risk of complications. In comparison to laparoscopic total hysterectomy, LASH is associated with decreased operating time and less urinary tract injuries. About 6% of the patients experience a cyclic bleeding of the cervical stump after LASH as a long-term complication [5]. A subsequent excision of the cervical stump is associated with a high risk of complications such as perioperative bleeding and gastrointestinal injuries [10].

LASH carries a low risk of developing a carcinoma of the cervical stump. According to the literature, it is a rare complication which occurs only in 0.3% of patients who undergo a cervix-sparing hysterectomy [11]. Most of patients who develop cervical stump carcinoma present with vaginal bleeding and discharge [12]. Surprisingly, a lot of women who underwent a LASH are not aware of the cervical cancer screening recommendations following the operation. According to Mattingly et al. [13], only 67% of the women were able to determine correctly postoperatively if the cervix was removed during surgery, 59% were aware that PAP Test screens and prevents cervical cancer and only 40% knew that HPV is associated with cervical cancer, underlining the urgent need to improve patient counseling preoperatively [11, 13]. Moreover, LASH should not be recommended in women who have a high risk of developing a cervical carcinoma, such as abnormal PAP smears or infection with high-risk of HPV. It has been suggested that electrocoagulation of the cervical mucosa during a supracervical hysterectomy may reduce the risk of developing a carcinoma of the uterine stump, a procedure which is commonly used to prevent cyclic bleeding [14].

Upon suspicion of cervical carcinoma, diagnostic investigations include clinical examination with biopsy, transvaginal sonography, CT scan and MRI of the pelvis. CT scan provides the oncologist with information about distant metastases, whereas MRI is fundamental for assessing local and regional invasion.

Surgical treatment of cervical squamous cell carcinoma changed dramatically after the results of the LACC trial in 2018 [15]. Before the publication of that study, a laparoscopic radical hysterectomy with pelvic node dissection was the gold standard for early-stage cervical cancers. After highlighting that minimally invasive approach correlates with increased risk for tumor recurrence and death in comparison to an open radical hysterectomy for early stage cervical cancers with no differences in intra-operative and post-operative complication rates, LACC trial subverted current knowledge and changed the standard of care [15, 16]. Data regarding 90-day treatment-related morbidity after the shift from laparoscopic approach to open surgery in referral centers showed no difference between the groups [17]. Regarding the treatment of early-stage adenocarcinoma of the cervix, there is need for further studies to investigate the role of minimal invasive surgery in this subgroup [18].

Therapeutic strategies and overall prognosis do not differ to those with an intact uterus, although it is suggested that early-stage carcinoma of the cervical stump is found rarer compared to cervix carcinoma of an intact uterus [19]. Thus, it can be presumed that cervical stump cancers have a poorer prognosis due to higher stage at diagnosis compared to cervical cancer cases in patients with intact uterus [20]. Hellström et al. [21] reported no poorer prognosis after radiologically treated squamous cervix carcinoma of the uterine stump compared to patients with intact uterus with an increased complication rate in stump cancer cases, presumably due to anatomic modifications in the pelvis following a LASH [21]. Extended surgery consisting of radical vaginal trachelectomy, abdominal radical trachelectomy, radical parametrectomy, exenteration or most commonly radiation or combined radiochemotherapy depending on tumor stage are the principles of treating cervical stump carcinoma [22–25].

After detailed and lengthy discussion with the patient of the possible risks of each treatment modality, the decision was made in a multidisciplinary setting to perform a staging lymphadenectomy with adjuvant radiochemotherapy according to German and international guidelines. Unfortunately, the patient suffered a tumor recurrence 5 months after the initial treatment with distant pulmonary and pleural metastases. She is now being treated in a palliative setting with a combined multichemotherapy and immunotherapy.

In conclusion, benefits of supracervical hysterectomy should be weighted against potential risks of this surgical procedure, such as developing a carcinoma of the cervical stump. Patients should be educated properly, particularly about the mandatory future cervical cancer screening following surgery. Preoperative counseling of patients is a fundamental responsibility of surgeons and regular training is required in order to inform the patient properly. After development of cervical stump carcinoma, the therapeutic approach does not differ from that of a cervical carcinoma with an intact uterus, although stump cancer cases seem to be diagnosed in a more advanced stage compared to cancer cases in intact uterus.

## Data Availability

The clinical data used to support the findings of this study are stored at Department of Gynecology, Obstetrics, Gynecological Oncology and Senology, Academic Teaching Hospital, Bethesda Hospital, Duisburg, Germany and are available from the corresponding author upon request.

## Abbreviations

LASH: Laparoscopic Supracervical Hysterectomy
PAP: Papanicolaou
TNM: Tumor, Node, Metastasis
FIGO: International Federation of Gynaecology and Obstetrics
CT: Computed Tomography
MRI: Magnetic Resonance Imaging
SI: Small Intestines
CS: Cervical Stump
IVC: Inferior Vena Cava
AB: Aortic Bifurcation
PDL-1: Programmed Death Ligand-1
